# Microfluidic Gas Sensors: Detection Principle and Applications

**DOI:** 10.3390/mi13101716

**Published:** 2022-10-11

**Authors:** Sreerag Kaaliveetil, Juliana Yang, Saud Alssaidy, Zhenglong Li, Yu-Hsuan Cheng, Niranjan Haridas Menon, Charmi Chande, Sagnik Basuray

**Affiliations:** 1 Department of Chemical and Materials Engineering, New Jersey Institute of Technology, Newark, NJ 07102, USA; 2 Department of Biomedical Engineering, New Jersey Institute of Technology, Newark, NJ 07102, USA

**Keywords:** microfluidics, gas sensing, sensitivity, selectivity

## Abstract

With the rapid growth of emerging point-of-use (POU)/point-of-care (POC) detection technologies, miniaturized sensors for the real-time detection of gases and airborne pathogens have become essential to fight pollution, emerging contaminants, and pandemics. However, the low-cost development of miniaturized gas sensors without compromising selectivity, sensitivity, and response time remains challenging. Microfluidics is a promising technology that has been exploited for decades to overcome such limitations, making it an excellent candidate for POU/POC. However, microfluidic-based gas sensors remain a nascent field. In this review, the evolution of microfluidic gas sensors from basic electronic techniques to more advanced optical techniques such as surface-enhanced Raman spectroscopy to detect analytes is documented in detail. This paper focuses on the various detection methodologies used in microfluidic-based devices for detecting gases and airborne pathogens. Non-continuous microfluidic devices such as bubble/droplet-based microfluidics technology that have been employed to detect gases and airborne pathogens are also discussed. The selectivity, sensitivity, advantages/disadvantages vis-a-vis response time, and fabrication costs for all the microfluidic sensors are tabulated. The microfluidic sensors are grouped based on the target moiety, such as air pollutants such as carbon monoxide and nitrogen oxides, and airborne pathogens such as *E. coli* and SARS-CoV-2. The possible application scenarios for the various microfluidic devices are critically examined.

## 1. Introduction

The rising air pollution and the onset of the pandemic have accelerated the need to produce sensors that can detect gases and airborne pathogens with high selectivity and sensitivity. However, the conventional sensors and methods to detect gases and airborne pathogens cannot be used for point-of-use applications. Developing sensors for point-of-use (POU) and point-of-care (POC) applications remains challenging because of various limitations. An ideal sensor for POU applications should be portable, low-cost, and able to detect target analytes in real-time with high specificity and sensitivity. Miniaturized sensors are essential in applications such as analyzing low amounts of toxic gases in industrial sites, low concentrations of biomarker gases in breath, gases released from food to test its freshness, analyzing the concentration of particulate matter in the atmosphere, and analyzing airborne viruses and bacteria. Gases such as ammonia, hydrogen peroxide, and volatile organic compounds (VOCs) cause adverse effects on human health and the environment and needs to be monitored [1]. There should be portable sensors that could detect airborne pathogens such as SARS-CoV-2, responsible for the recent pandemic and death of millions of people. Developing miniaturized gas sensors for detecting the low volume of biomarker gases, such as dimethysulphide—a biomarker for liver disease, and ketones—a biomarker for diabetes, will lead to a new era of non-invasive diagnostics [2].

Microfluidics is an emerging technology and an excellent tool for developing miniaturized sensors. There are various advantages of integrating microfluidics with gas sensors. In the area of sensors, microfluidics technology allows the analysis of a small volume of samples. It allows the use of a small volume of reagent or sensing material. Furthermore, the ability to analyze a small sample volume translates into a sensor with a quick response and low sample volume requirement [3].

Furthermore, microfluidics allows the channeling of the flow of gases to the sensing material. In this process, the gas parameters such as volume and flow rates can be controlled, making the results reproducible [4]. Microfluidics devices can be classified into two categories continuous-flow microfluidics and droplet-based microfluidics. In continuous-flow microfluidics, the fluid is flowed through the channel and is manipulated without breaking the continuity. Droplet-based microfluidics deals with creating droplets in the channels using two or more immiscible fluids at a T-junction [5]. Both classes of microfluidics have been used to develop gas sensors.

In the early 2000s, microfluidics was integrated with metal oxide semiconductors (MOS) to develop a miniaturized gas sensor. The goal is to develop sensors that can analyze the small volume of gases. In one of the first papers that used microfluidics for gas sensing, microfluidic channels are integrated with a tin-oxide gas sensing layer to develop a miniaturized gas sensor [6]. Further development happened when microfluidic channels with different coating materials were used to introduce selectivity to MOS-based gas sensors. This idea helped researchers to develop a MOS-based gas sensor that could differentiate between different volatile organic compounds [7]. Recently field effect transistors (FETs) functionalized with recognition molecules are integrated with microfluidic platforms to detect gases such as trimethylamine (TMA) [8] and airborne pathogens such as Influenza A(H3N2) [9]. The latest development in microfluidic gas sensors uses optical techniques such as surface-enhanced Raman spectroscopy and fluorescence to detect multiple analytes with high sensitivity and selectivity.

This review paper will give readers a comprehensive idea of the developments in microfluidic sensors for detecting gases and airborne pathogens. This review paper is structured in the following sequence. After the introduction, a review of microfluidic channel parameters explains different coating materials, geometry, and surface area enhancing features used in microfluidic platforms to improve detection efficiency. The subsequent section explains the different fabrication methods used to create microfluidic platforms. The following section, which is the main focus of this review paper, explains the different detection techniques used in continuous-flow microfluidic sensors. Non-continuous microfluidics, i.e., the bubble/droplet-based microfluidics used to detect gases and airborne pathogens, are described with microfluidic sensors developed and categorized based on applications explained next. Finally, the strategies to develop a complete microfluidic sensor are also elucidated. This review paper also discusses the merits and demerits of the existing microfluidic gas and airborne pathogen sensors, which show readers the possible future developments of microfluidic-based gas sensors.

## 2. Microfluidic Channel Parameters

The three critical parameters for developing a microfluidic gas sensor are coatings, geometry, and surface area of microfluidic channels.

### 2.1. Microfluidic Channel Coatings

Device coatings are usually used in microfluidic devices that use MOS as the sensing layer. The gases diffuse through the microfluidic channel before interacting with the MOS layer. Coating the microfluidic channel with polymers brings some discrimination ability to MOS-based sensors, which otherwise produce almost the same transient response to different gases [10]. Distinct transient response is due to the difference in the physical adsorption/desorption rates of different gas molecules to/from the channel.

**Figure 1:**
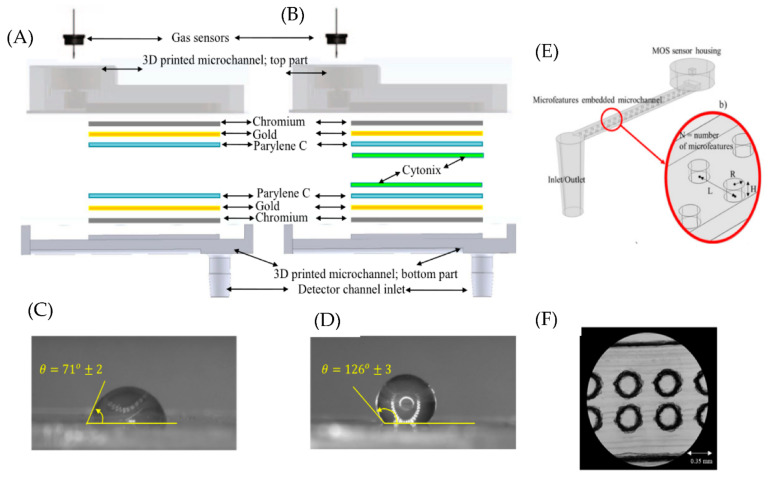
(**A**) Schematic of microfluidic channel with chromium, gold and Parylene C coating. (**B**) Schematic of microfluidic channel with chromium, gold, Parylene C and Cytonix coating. (**C**,**D**) Contact angle values for DI water on coating without and with cytonix. (**E**) Schematic of microfluidic channel with microfeatures. (**F**) Microscopic image of microfeatures. (**A**–**D**) Reproduced under the terms of CC BY 4.0 license from [12] Copyright (2019), The Authors, published by Nature. (**E**,**F**) Reprinted from [16], Copyright (2022), with permission from Elsevier.

Polymer coatings such as Poly(3,4-ethylene dioxythiophene): Poly(styrene sulfonate) (PEDOT: PSS), Parylene C, and Cytonix are used in microfluidic channels to integrate with MOS sensors. PEDOT: PSS is a conducting polymer that forms a hydrogen bond with methanol and ethanol and shows strong adsorption to ketones due to the dipole-dipole forces [11]. Therefore, the microfluidic sensors coated with PEDOT: PSS can filter out methanol, ethanol, and ketones but detect gases such as carbon monoxide (CO), hexane, and benzene. Multilayer coatings are also used to improve the discrimination ability of MOS sensors. For example, microfluidic channels coated with Cr, Au (bottom layer), and Parylene C (top layer) can show rapid response and outstanding discrimination ability between ethanol, methanol, and acetone [10]. In follow-up work, it is found that devices coated with Cr, Au, Parylene C, and Cytonix (Figure 1A) show better discrimination ability than channels coated with Cr, Au, and Parylene C (Figure 1B) for different volatile organic compounds(VOCs) (methanol, ethanol, 1-propanol, 2-pentanol, acetone, pentane, and hexane) [12]. This is due to Cytonix being a fluoropolymer with excellent hydrophobic and oleophobic properties. This can be seen from the contact angle measurement shown in Figure 1C,D. This shows that the hydrophobicity of the microfluidic channel is an important parameter (appropriate coatings can modify that) that affects the selectivity in MOS-based microfluidic sensors.

### 2.2. Geometry and Dimension of Microfluidic Channels

Optimizing the micro-channel dimension is necessary to achieve good sensitivity, selectivity, rapid response, and low recovery time. For microfluidic sensors that use coating materials to introduce a discrimination ability, physical adsorption/desorption of the gases to the microfluidic channel is an important parameter to ensure good selectivity. In this context, channels with smaller depths are preferred to differentiate gases with similar diffusion coefficients since decreasing the channel depth can increase physical adsorption [10]. In addition, the channel length increases the sensor’s selectivity as it slows down the diffusion process. However, longer channels result in higher recovery times [10]. Hence it is essential to find the optimum channel dimension for microfluidic sensors.

Selecting a suitable channel shape is vital in increasing the sensitivity of microfluidic sensors. For example, Zhu et al. used a serpentine channel in their microfluidic photoionization detector to decrease the ionization chamber volume and eliminate dead volume while maintaining a large UV illumination area [13]. The result is a direct translation into a faster response time. Therefore, channel geometry becomes much more critical for sensors with integrated microfluidic air sampling platforms. In such cases, the channel’s geometry determines the analyte’s collection efficiency, which will determine the microfluidic system’s detection efficiency. For example, Jing et al. used a microfluidic channel with a staggered herringbone mixer structure for bacteria capture with a collection efficiency close to 100% [14]. This microfluidic chip is then coupled with their microfluidic sensor to detect a low concentration of bacteria.

### 2.3. Surface Area of the Microfluidic Channel

Another essential feature of a microfluidic gas sensor is the surface area of the microfluidic channel. In devices that use a sensing material in the microfluidic channel, it is essential to increase the gas molecules’ interaction with the channel material to increase the sensitivity of the microfluidic device. Sensitivity can be optimized by increasing the surface area of the microfluidic channel in which the sensing material/coating is applied. The obvious way to do this is by decreasing the dimensions of the microfluidic channel, as discussed in the previous section. Another interesting way is to incorporate micro and nano features along the microfluidic channel. Yang et al. reported using a microfluidic channel with a micro-structured triangular array coated with the sensing material [15]. The device detected multiple VOCs with a limit of 1 ppb. Increasing the surface area of the microfluidic channel can also increase the sensor’s selectivity. Ghazi et al. reported using microfluidic channels with micro and nanofeatures and a MOS sensor [16]. The schematic and the microscopic image of these microfeatures are shown in Figure 1E,F. The selectivity of the microfluidic device with micro and nanofeatures increased by 120% compared to a plain microfluidic channel.

## 3. Fabrication Materials and Methods

The most common material for microfluidic gas sensors is Polydimethylsiloxane (PDMS) because it can be fabricated easily and have well-known physical and chemical properties. Briefly, in PDMS, a mold is prepared using lithography. The PDMS, mixed with a curing agent, is poured onto the patterned mold and heated for several hours. The PDMS layer is peeled off from the master mold and is now ready for use. Laser engraving is another technique used to create microchannels in Polymethyl methacrylate (PMMA) substrate [11]. This is usually accomplished using a carbon dioxide (CO_2_) laser, breaking the bond in the polymer surface and removing the decomposed fragments from the ablation regions [17]. Conventional computer numerical control (CNC) machining has also been used to create microfluidic channels for the acrylic substrate [18]. Additive manufacturing techniques such as 3D printing are common ways to create a microfluidic device. Researchers have used 3D modeling and printing to design and manufacture channels with microfeatures [16].

Recently there have been developments in paper-based microfluidics for gas sensing. One of the easiest ways to prepare paper microfluidics is through wax printing. Wax is used to define the microfluidic channel and restrict the flow of liquids. The channel is fabricated by applying solid wax to the paper substrate or by melting solid wax into the paper substrate using a hot plate. Another way of creating paper microfluidics is by the ultraviolet (UV) curing method. The paper immersed in precursor solution is placed under a photomask and cured by a UV lamp. The treated paper is then transferred for pattern development using acetone. Sun et al. used this process to make 48 chips in an A4-size filter paper within 30 s at a total cost of 1.92$ [19].

These fabricated microfluidic platforms are used as gas sesnors by integrating different detection techniques. The merits and demerits of the exisiting microfluidics based gas sensors are listed in Table 1 (continuous flow microfluidics) and Table 2 (bubble/droplet based microfluidics).

## 4. Detection Approaches

Based on the transduction method used to detect the target analytes, detection approaches can be divided into three main categories, which are electronic, electrochemical, and optical methods. It is worth noting that there is a preferred application field for every detection approach.

### 4.1. Electronic

In electronic transduction, changes in resistance or conductivity of the sensing material are used to sense the presence of target analytes. Electronic gas sensors can be divided into two primary categories MOS and field effect transistor-based gas sensors.

#### 4.1.1. Metal Oxide Semiconductor

MOS is one of the most used sensing materials in gas sensors. MOS was first used as a gas sensor in 1952 when it was demonstrated that some semiconductor materials modify their resistance depending on the surrounding atmosphere [39]. The adsorption of gases onto MOS leads to a change in majority charge carrier concentration which in turn changes the material’s resistance [40]. In the case of an n-type MOS, interaction with reducing gases decreases the resistance, while interaction with oxidizing gases leads to an increase in resistance [41]. Just the opposite happens in the case of a p-type MOS sensor. A more detailed working principle of the MOS gas sensor is explained in this reference [40]. There are many reports of integrating MOS sensors with the microfluidic device to realize a miniaturized gas sensor [4,11,12,16]. Martini et al. developed a microfluidic gas sensor with an integrated pumping system to detect ammonia [4]. The microfluidic channel’s end consists of a tungsten trioxide-sensitive layer with interdigitated platinum electrodes and an integrated heater. The heater serves a dual purpose (i) to maintain the temperature of the metal oxide sensing layer and (ii) to maintain a temperature gradient along the microchannel. The temperature gradient ensures gas flow through the microfluidic channel, called the thermal creep phenomenon. The MOS sensing layer needs to be maintained at a specific temperature to ensure reproducible results. An optimum working temperature of 473 K is maintained using the heater. This sensor’s linear range of detection is between 10 to 100 ppm. The sensor’s response time is around 10 min, showing reversibility, reproducibility, and baseline stability.

**Figure 2:**
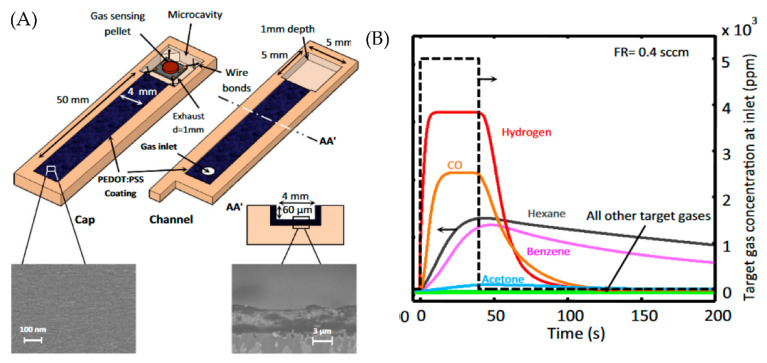
(**A**) Schematic representation of PEDOT: PSS-coated microfluidic channel integrated with gas sensor (**B**) Temporal response of the sensor to the target gas along the microchannels coated with PEDOT: PSS. Reproduced under the terms of CC BY 4.0 license from [11] Copyright 2017, The Authors, published by Nature.

However, one of the significant drawbacks of the MOS sensors is poor selectivity. In the case of an n-type MOS sensor, in general, interaction with any of the reducing gas (H_2_, NH_3_, CO, and VOCs [42]) decreases the resistance and interaction with any of the oxidizing gas (O_3_, O_2_, NO_2_ and Cl_2_ [43]) increase the resistance. To increase the selectivity of MOS sensors, many researchers have integrated microfluidic channels with different coating materials [11,12,16]. Each gas produces a distinct temporal response (as shown in Figure 2B) due to interaction with the channel coating, thereby introducing discrimination ability for the MOS sensor. For example, Ghazi et al. fabricated a Parylene C-coated microfluidic channel with microfeatures (by 3D printing) and nanofeatures (by Graphene oxide). They integrated it into a MOS sensor to detect various VOCs [16]. As a result, 6 different VOCs are passed individually through the microfluidic channel and exposed to the MOS sensor at the end of the microfluidic device. The MOS sensor’s resistance change is used to detect the presence of gases. When different gases interact with the channel coating, depending on the properties of the gas, the rate of adsorption/desorption and the diffusion rate will be different, producing a distinct temporal response for each gas. The principal component analysis is used to quantify the changes in the dynamic response of different gases. The selectivity of the microfluidic gas sensor embedded with cylindrical microfeatures increased by 64.4% (as compared to the plane channel). The selectivity of the microfluidic gas sensor with both cylindrical microfeatures and nanofeatures increased by 120.9%. This shows the importance of increasing the surface area of the microfluidic channels.

Hossein-Babaei et al. used a microfluidic channel coated with PEDOT: PSS for the selective detection of volatile organic compounds and carbon monoxide [11]. A MOS sensor is located at the end of the microfluidic channel, as shown in Figure 2A. The sensor allowed the passage of hexane, benzene, and CO but blocked the passage of methanol, ethanol, and acetone. This response is shown in Figure 2B. This selective filtration is due to the formation of hydrogen bonding between the molecules and the coated channel walls. Methanol and ethanol form hydrogen bonds with oxygen atoms of the PEDOT macromolecular chain, while acetone accepts hydrogen bonding from the sulfonate group. While carbon monoxide, benzene, and hexane cannot participate in the hydrogen bonding since their molecular dipole is either negligible or zero.

Even though MOS sensors integrated with a microfluidic channel are excellent at discriminating between different pure VOCs, MOS-based sensors still cannot selectively detect gases in a gas mixture. It is also incapable of separating different gases in space and time, as is accomplished using a conventional gas chromatography system. These limitations mean that MOS sensors coupled with microfluidic platforms cannot be used as a standalone system to detect different gases in a gas mixture.

#### 4.1.2. Field-Effect transistors (FET)

**Figure 3:**
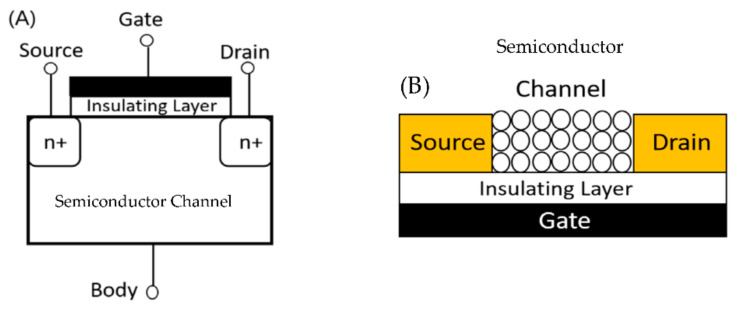
(**A**) Structure of FET (**B**) Modified FET used for gas sensing.

FET consists of a source electrode, a drain electrode, a gate electrode, a semiconductive material channel, and an insulating gate oxide [44]. The schematic of FET is shown in Figure 3A. In a FET, voltage is applied at the gate electrode, which changes the conductivity of the channel semiconductor, changing the current flowing from source to drain. There are mainly two ways of using FETs as a sensor for air. (1) Conventional FET can detect charged aerosols by making them adsorb on the gate of FET. The adsorbed charged particles will act as an extra virtual gate bias and change the current value of FET [20]. (2) To detect specific molecules (target), the channel semiconductor (as shown in Figure 3B) or the gate is functionalized with a recognition molecule. When the surface is exposed to target analytes, the specific binding will lead to a change in conductance or surface potential [8,9,21]. Generally, nanowires are used as semiconductors in such scenarios because of their high surface-to-volume ratio.

**Figure 4:**
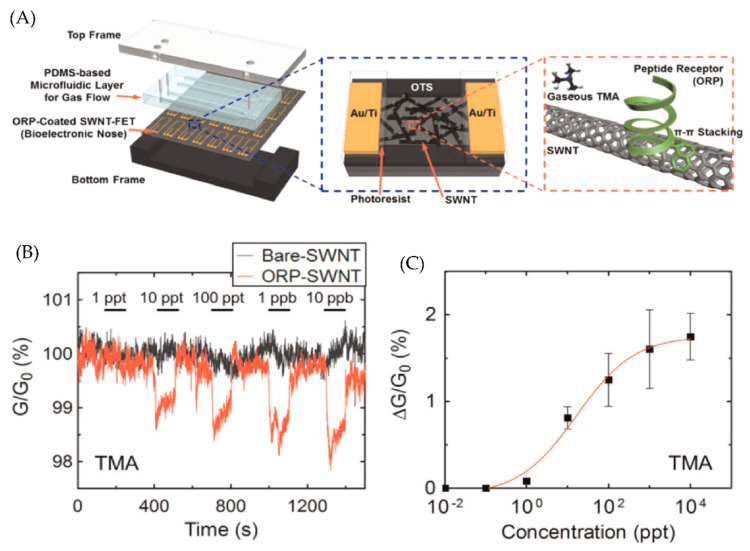
(**A**) Schematic representation of bioelectronic nose (**B**) Real−time measurements of change in conductance when Bare and ORP coated SWNT is exposed to TMA. (**C**) Calibration curve of the bioelectronic nose. Reprinted from [8], Copyright (2015), with permission from Elsevier.

Lee et al. combined a bioelectronic nose with a microfluidic system to detect gaseous TMA in real-time [8]. Single-walled carbon nanotube-field effect transistors (SWNT-FETs) are used as a sensor in the microfluidic system. SWNT-FETs are functionalized with olfactory receptor-derived peptides (ORPs) that can recognize the TMA molecule. The fabricated sensor consists of two layers, (i) a PDMS-based microfluidic channel for gas flow and (ii) ORP-coated SWNT-FETs, sandwiched between a top and bottom frame. The schematic of the fabricated device is shown in Figure 4A. A change in the conductance of the ORPs-coated SWNT-FETs is used as the signal to create a calibration curve, as shown in Figure 4C. The limit of detection (LOD) for this gas sensor is as low as 10 ppt.

Kuznetsov et al. fabricated a bioelectronic nose using an ion-selective field effect transistor (ISFET) to detect vanillin in the gas sample [21]. In an ISFET, the metal gate is replaced by an ion-selective membrane, an electrolyte, and a reference electrode [45]. Aptamers are immobilized on the gate surface to detect vanillin selectively. The gas sample can interact with the electrolyte through pores in the sensor wall with a hydrophilic microfluidic channel. The change in the surface potential of ISFETs when exposed to different concentrations of vanillin gas samples is used to create the calibration curve. An increase in vanillin concentration led to an increase in the surface potential. The fabricated device showed a limit of detection of 2.7 ppt and linear response in the range of 2.7 ppt to 0.3 ppm. In a selectivity study, the sensor response to other odor molecules is analyzed, and the response generated by gaseous vanillin is much stronger than that generated by other odorant molecules.

FET-based microfluidic sensors are also ideal for detecting airborne pathogens. Shen et al. developed a real-time monitoring system for airborne influenza H3N2 viruses by integrating silicon nanowire FET (SiNW FET), microfluidics, and bioaerosol-to-hydrosol air sampling technique [9]. The surface of the p-type SiNW is functionalized using Influenza A (H3N2) subtype antibody. The binding of the H3N2 virus with the antibody increased the conductance. Higher virus concentrations in air samples corresponded to higher conductance levels in the SiNW devices. The selectivity of this device is successfully demonstrated using clean, indoor air samples and Der p1 allergens.

Yanna et al. have developed an extended-gate field-effect transistor (EGFET) for fine particulate matter (PM) detection integrated with a virtual impactor made of microchannels to separate the fine and coarse particles [20]. The fine particles are deposited on a water-soluble gelatin membrane by thermophoresis. The gelatin membrane is transferred and dissolved in a liquid chamber. This solution is added to the PM detection system, in which a gold extended electrode is connected to the gate terminal of a conventional metal-oxide-semiconductor field-effect transistor. Since the particles in the solution are charged, the adsorption of particles onto the gold electrode functions as an extra virtual gate bias, increasing the FET’s current value. Different sizes of PM (PS2—2 μm, PS5—5 μm, PS10—10 μm) corresponded to different levels of current flow in the device. The exciting aspect of this work is that the sensor can differentiate between different-sized particles.

### 4.2. Electrochemical

Electrochemical sensors have shown rapid response, high sensitivity, and low cost and can be readily used for POU applications [46]. Voltammetry, amperometry, and electrochemical impedance spectroscopy (EIS) are the most used analytical tools to probe the electrochemical system. Nevertheless, in the field of microfluidic gas sensors, these techniques are under-utilized. Generally, in an electrochemical sensor, the current produced by redox reactions involving the target analyte is used as the signal to detect and quantify the amount of the target analyte. In non-faradaic electrochemical sensors, where there is no redox reaction, changes in the dielectric properties due to the binding interaction between the target and probe are used as the sensing signal [47].

#### Amperometry

In amperometric detectors, the change in the current is measured with time while the constant potential is maintained. Therefore, Amperometry is an excellent detection technique for developing real-time sensors. Cha et Al. developed an amperometric gas sensor for the real-time detection of nitric oxide (NO) in cell culture present within a microfluidic device [22]. As the working electrode, the sensor is directly integrated with a gold/indium-tin-oxide electrode on a porous polymer membrane(pAu/ITO). Au-hexacyanoferrate (Au-HCF) layer is electrochemically deposited on pAu/ITO. Au-HCF is used to catalyze the NO oxidation to nitrite. The developed gas sensor can be integrated as a detector within a non-PDMS microfluidic device. The real-time sensing capability of the NO sensor is studied by injecting different concentrations of NO stock solution into the microfluidic channel and then recording the current response. The selectivity is achieved using a selective gas permeable membrane (TeflonAF-treated Celgard membrane), which allows nitric oxide but prevents other oxidizable interfering agents such as ammonia. The LOD of the sensor is reported as 1 nM. Extracellular NO concentration from a small number of macrophage-type cells (stimulated by an endotoxin (lipopolysaccharide)) is detected using the developed NO detector. The detection of NO from a small number of cells is made possible due to the integration of the NO sensor with microchannels.

**Figure 5:**
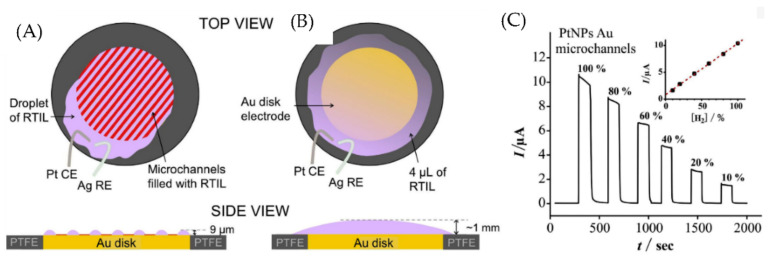
Illustration of (**A**) Au microchannel electrode and (**B**) Au macrodisk electrode. (**C**) Long-term chronoamperometry for 10–100% vol H_2_ PtNP modified Au microchannel. Reprinted from [23], Copyright (2019), with permission from Elsevier.

Hussain et al. used a platinum nanoparticle (PtNP) modified gold microchannel array as a new electrode type for real-time detection of hydrogen [23]. Modification of gold microchannel with PtNP allows the reversible oxidation of hydrogen. Room temperature ionic liquid (RTIL) 1-ethyl-3-methylimidazolium bis(trifluoromethylsulfonyl)imide ([C_2_mim][NTf_2_]) is filled in the microchannel for use as electrolyte. RTILs are salts that have a melting point below room temperature. They have near zero-vapor pressure, which makes them an ideal candidate for use as very thin electrolyte layers. Long-term chronoamperometry results showed better sensitivity (3.4 vol%) and ultra-fast response times (2s) for PtNP-modified Au microchannels compared to PtNP-modified Au microdisks. The fast response time and high sensitivity of PtNP-modified Au microchannels compared to PtNP-modified Au microdisks are attributed to the use of microchannels with a very thin layer of ionic liquid (as shown in Figure 5). The smaller thickness of the microchannel results in a smaller diffusion distance for gases to reach the electrode, reflecting a faster response time.

It is worth noting that electrochemical techniques are under-utilized in the development of microfluidic gas sensors. One of the main reasons for this could be the need for a reversible electrochemical reaction. For developing a faradaic electrochemical gas sensor for real-time continuous sensing, the electrochemical reaction must be reversible and preferably a single-step redox reaction [48]. If the reaction is irreversible, the products of the reaction might poison the electrode or electrolyte. Therefore, non-faradaic electrochemical techniques such as non-faradaic EIS become essential. EIS is an extremely sensitive technique that can detect changes in the electrode-electrolyte interface to confirm the presence of some impurity (target analyte). Due to its high sensitivity, this technique is extensively used in sensors for detecting biomolecules [49] and water contaminants such as perfluorooctanesulfonate [50]. Unfortunately, this technique is yet to be incorporated with microfluidic gas sensors.

### 4.3. Optical

Optical gas sensors rely on the change in the absorbance/color [18,19,24,34], luminescence [25,26,27,28], or change in the Raman signature [15,31,32,33] of the sensing material to detect the presence of the target analyte. Results obtained from some of the optical sensors can be seen by the naked eye, which allows for the easy miniaturization of the sensor since it does not require any external device or power supply.

#### 4.3.1. Colorimetric

In the colorimetric technique reaction of the target analyte with a specific reagent causes a color change. The intensity of the color is directly proportional to the concentration of the analyte in the sample. This technique is instrumental in developing low-cost disposable gas sensors. It is also valuable for applications where quantifying the target analyte is unnecessary—where the user is looking for the presence or absence of the target analyte.

**Figure 6:**
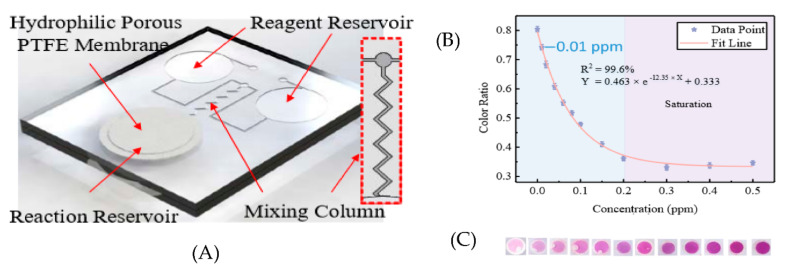
(**A**) Schematic of the microfluidic platform (**B**) Calibration curve of microfluidic colorimetric sensor (**C**) reaction reservoir images when exposed to different concentration of gaseous formaldehyde. Reproduced under the terms of CC BY 4.0 license from [24] Copyright 2018, The Authors, published by MDPI.

Guo et al. designed a microfluidic colorimetric sensor that detects gaseous formaldehyde [24]. The microfluidic chip consists of two reagent reservoirs, a reaction reservoir, and a mixing column—4-aminohydrazine-5-mercapto-1,2,4-triazole (AHMT) solution and potassium hydroxide solution filled reagent reservoirs 1 and 2, respectively. The mixture of reagents 1 and 2 will react with formaldehyde and change the color of the solution to purple. Different shades of purple relate to different concentrations of absorbed formaldehyde, as shown in Figure 6C. The reaction reservoir is covered with a hydrophobic porous PTFE membrane that allows the sample gas to react with the reagent. The schematic of the microfluidic platform is shown in Figure 6A. A smartphone captured the solution’s image and determined the analyte concentration with an extremely low LOD of 0.01 ppm. The calibration curve is shown in Figure 6B. The sensor can be used as a disposable gas sensor to detect formaldehyde.

The apparent limitation of a colorimetric technique is that once the reagent is consumed, either the sensing layer should be changed or a new sensor should be used. To overcome this limitation, Wan et al. used a microfluidic channel with the sensing material present throughout the channel [18]. O-phenylenediamine (PDA) is used as the sensing material. PDA is colorless and turns yellow upon reaction with nitrogen dioxide (NO_2_). PDA offers excellent selectivity against other common gases present in ambient air. The sample air flows through this sensor’s microfluidic channel coated with PDA. The reaction of the NO_2_ with PDA changes the color along the microfluidic channel, which a CMOS imager monitors in real-time. An image processing routine is used to analyze the color gradient along the channel and determine the analyte concentration. Color in the region near the inlet changes first, as it consumes most of the analytes. There is fresh sensing material downstream of the channel, which enables continuous detection. The developed sensor could continuously monitor air quality over 18 h with a detection limit of 50 parts per billion per volume (ppbV).

Paper is one of the ideal substrates used with the colorimetric technique. Therefore, a paper-based colorimetric sensor is ideal for use as a disposable sensor. Sun et al. used paper-based microfluidics to detect different airborne trace metals [19]. The fabricated paper microfluidic chip consists of 12 detection reservoirs and a circular central inlet to introduce the sample. 6 different colorimetric ligands chrysoidine-G, dithiooxamide, 1,10phenanthroline monohydrate, 4-(2-pyridylazo) resorcinol, 1,5-diphenylcarbazide and dimethylglyoxime (dmg) corresponding to Co, Cu, Fe, Mn, Cr, and Ni is used as the metal assays. The selected ligands can chelate to metal ions resulting in the distinct coloration associated with different metals. The LOD of the sensor is 8.16, 45.84, 186, 10.08, 152, and 80.40 ng for Co, Cu, Fe, Mn, Cr, and Ni, respectively.

#### 4.3.2. Surface-Enhanced Raman Spectroscopy (SERS)

Raman spectroscopy measures the fingerprint vibrational spectrum of the target analytes. However, due to the low intensity of the Raman signal, it cannot be used as a detection technique to develop sensors with high sensitivity. SERS is the enhancement in the Raman signal achieved when the molecules are adsorbed on a noble metal surface [51]. It has achieved the ultimate detection limit, the detection of a single molecule [52,53].

The SERS technique was first integrated with microfluidics to detect 4-aminobenezenethiol(4-ABT) [29]. Piorek et al. used a microfluidic channel filled with a colloidal suspension of silver nanoparticles open to the atmosphere. The surface tension at the free-surface interface is used to confine the flow of the colloidal solution (at 60 μm/s) through the microchannel. This SERS active colloidal solution flow through the microchannel provides a continually refreshing SERS substrate. When 4-ABT is exposed to the colloidal solution, it binds to the nanoparticles, initiating an aggregation process. The formation of dimers (two nanoparticles bound to a single adsorbate) produces the maximal SERS intensity at 1435 cm^−1^ (Raman band of 4-ABT), which happens between a length of 50 to 150 μm in the microchannel. Furthermore, an exposure time of ≈1 to ≈3 s is enough to generate maximal SERS intensity when exposed to gaseous ≈ 300 uM 4-ABT. The developed sensor can is ideal for continuous real-time detection of 4-ABT. In follow-up work, the research group used the fundamental principles of the previous sensor and developed a free-surface microfluidic device for detecting 2,4-dinitrotoluene (2,4-DNT) [30]. Two distinct peaks appeared at 1350 cm^−1^ and 1600 cm^−1^ approximately 2 min after exposure to 1 ppb of 2,4-DNT.

One of the main advantages of the SERS technique is that it can be used for multiplex sensing. Yang et al. have developed multiple microfluidic sensors that use the SERS technique for multiplex sensing. In the first work, Yang et al. designed a microfluidic gas sensor to detect multiple aldehydes at extremely low concentrations [15]. A composite nanoparticle is fabricated in the form of a core-shell structure as the SERS probe. The SERS probe consists of a cubic silver nanoparticle as the core and a layer of metal-organic framework (MOF) material [Zeolitic Imidazolate framework-8 (ZIF-8)] as the shell. Cysteamine (CA), a gas-capturing agent selective to aldehydes, is embedded between the core and shell. The microfluidic channel consists of a micro-structured triangular array coated with the SERS probe. The triangular array is employed in the channel to increase the collision of gases to the SERS probe. The sensor detected benzaldehyde and 3-ethylbenzaldehyde with a concentration as low as 1 part per billion (ppb). The sensor could also simultaneously detect 3 different aldehydes (benzaldehyde, 3-ethylbenzaldehyde, and glutaraldehyde). This research shows that SERS is an excellent technique that could be employed to create gas sensors with high selectivity and multiplex sensing ability.

In follow-up work, Yang et al. developed a microfluidic gas sensor with an ordered 3D SERS substrate and an ultra-flexible Ti_3_C_2_T_x_ MXene for the multiplex detection of VOCs [33]. The 3D SERS substrate is composed of polystyrene microspheres with nano pits containing bimetallic nanocubes (gold core of 30 nm, silver nanocube of 54 nm, and a 2 nm gold shell). The microspheres prepared using colloidal self-assembly are arranged in an ordered honeycomb-such as structure, which solves the repeatability problem of SERS substrates. Ti_3_C_2_T_x_ MXene adheres to the 3D SERS substrate, which serves as a universal adsorption layer for various VOCs. 2,4-dinitrotoluene (DNT), odoriferous benzaldehyde, and indole are chosen as the VOCs to test the detection efficiency and multiplexing capability. The classical least square method differentiated the peaks corresponding to each VOC. The LOD for DNT, odoriferous benzaldehyde, and indole is 10, 10, and 50 ppb, respectively. In addition, a chromatic barcode is developed to read out the complex composition of the samples visually.

Lafuente et al. fabricated a microfluidic device to detect neurotoxic agents in the gas phase using the SERS technique [32]. The microfluidic channel is coated with gold-mesoporous silica NPs, which exhibit huge sorption capacity towards the gas phase and a remarkable SERS enhancement factor. This microfluidic device could detect dimethylmethlyphosphonate (DMMP) in the gas phase at 2.5 ppmV. Lee et al. used direct metal writing using two-photon lithography to create a microfluidic channel with densely packed gold nanoparticles [31]. The developed platform is used as a SERS detector for the real-time detection of the gaseous 4-MBT molecule. The versatility of the developed microfluidic device in detecting gases such as acetone and ethanol, which has no specific affinity to Au, is also demonstrated.

#### 4.3.3. Fluorescence

Fluorescence-based techniques are generally used for detecting bacteria and viruses. Three main techniques are generally used in fluorescence-based sensors. (1) The use of fluorescent dyes to stain bacteria [25]. Typical dyes used in such scenarios are cell permeable and show very high fluorescence when bound to DNA or RNA. (2) The use of antibody-conjugated fluorescent particles for detecting viruses [26]. (3) loop-mediated isothermal amplification technique (LAMP) to detect bacteria and viruses [28]. LAMP is a DNA amplification technique such as a polymerase chain reaction (PCR). Unsuch as PCR which uses 2 primers recognizing 2 regions of the target DNA, LAMP uses 4 primers recognizing 6 distinct regions of the target DNA, making it highly selective [54]. Moreover, the traditional PCR method requires heating and cooling cycles, while the LAMP technique uses isothermal conditions, making it suitable for implementing miniaturized sensors. Finally, such as a calcein, a dye binds with the LAMP products and gives out a fluorescent emission [55]. Microfluidic fluorescent sensors developed by these 3 techniques are discussed below.

Kim et al. developed a paper microfluidic chip to capture airborne droplets containing SARS-CoV-2 directly and used fluorescence for detection [26]. The microfluidic chip contains 4 dumbbell-shaped microfluidic channels. The solution containing human saliva with different concentrations of SARS-CoV-2 is sprayed 2 times (simulating typical human coughs) and 5 times (simulating repetitive human coughs) into a chamber containing the microfluidic chip. To detect the virus, antibody-conjugated sub-micron fluorescent particle suspension is added to the microfluidic channel, which induces antibody-antigen binding. Then, a smartphone-based fluorescence microscope was used to quantify the particle aggregation, which confirmed the presence of SARS-CoV-2 in the air. This entire process of virus collection to detection took about 30 min.

Jiang et al. reported using a microfluidic device to capture and enrich five airborne bacteria, followed by on-chip loop-mediated isothermal amplification (LAMP) for detecting the airborne bacteria [28]. A microfluidic device with a staggered herringbone mixer (SHM) structure is used to capture and enrich the airborne bacteria. The outlet of the capture chip is connected to the LAMP chip. The LAMP chip consists of 5 microfluidic channels, each connected to a reaction chamber. Each channel consists of LAMP primers targeting different bacteria (*S. aureus*, *E.coli*, *P. aeruginosa*, *C. koseri*, and *K. pneumonia*). The bacterial lysate is mixed with the LAMP reaction mixture (mainly containing polymerase and calcein dye) and injected into the microfluidic chip. A fluorescence signal (visible to the naked eye) is observed between the LAMP product and the calcein dye when a reaction occurs. The detection limit of the LAMP chip for *S. aureus* is found to be 24 CFU (colony-forming unit).

**Figure 7:**
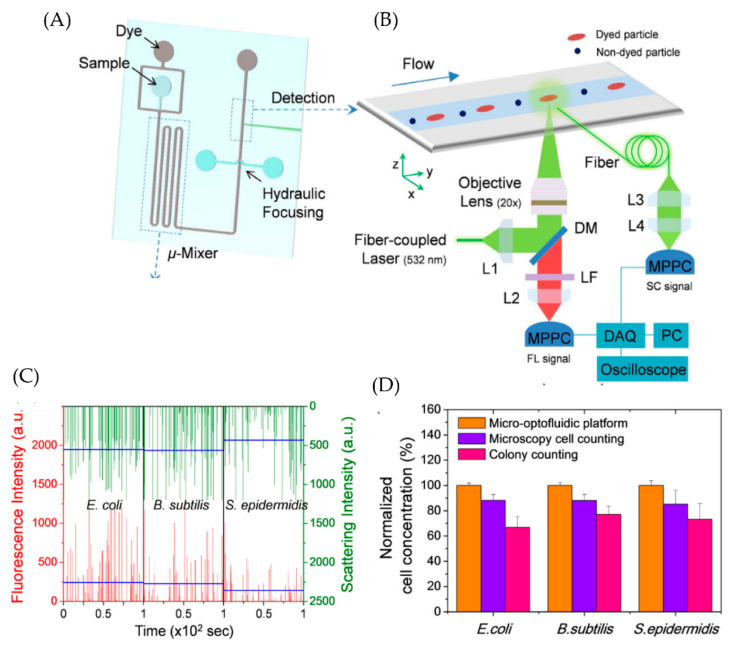
(**A**) A schematic diagram showing the structure of microfluidic platform (**B**) Optical setup for the real-time detection of the target analytes (**C**) Real-time fluorescence and scattering signal acquired for all three bacterial cells. Light scattering intensity shows the presence of non-bacterial cells (**D**) Bacterial cell concentrations measured using the micro-optofluidic platform, cell counting using fluorescence microscopy, and colony counting. Reproduced under the terms of CC BY 4.0 license from [25] Copyright 2015, The Authors, published by Nature.

The fluorescence-based microfluidic platforms discussed above are not ideal for continuous real-time detection. Therefore, Choi et al. developed a micro-optofluidic platform that uses fluorescence and light scattering to determine the airborne bacteria number concentration and residue particles in real-time and continuously [25]. The PDMS microfluidic platform consists of a sample and dye (SYT082 dye) reservoir, a micromixer for bacteria staining, and a detection component, as shown in Figure 7A. SYT082 provides cell-permeant nucleic acid staining and exhibits low fluorescence in a cell-free system [56]. Multipixel photon counter (MPCC) is used for continuous real-time detection of scattering light and fluorescence light from target particles, as shown in Figure 7B. Gram-negative *Escherichia coli*, Gram-positive *Bacillus,* and Gram-positive *Staphylococcus epidermidis* are chosen to test the real-time detection capabilities. The obtained results are shown in Figure 7C. The micro-optofluidic platform showed better detection efficiency than traditional microscopy cell counting and colony culture methods.

#### 4.3.4. Non-Traditional Methods

Jayan et al. developed an optical lab-on-a-chip device by integrating a ninhydrin-polymer composite into a microfluidic device to detect ammonia [34]. The chemisorption of ammonia onto the composite resulted in a change in the optical absorption property. The lab-on-a-chip device has an integrated light-emitting diode and photoresistor, enabling the detection and quantification of ammonia. The LOD of this sensor is as low as 2 ppm.

Zhu et al. designed a microfluidic photoionization detector (PID) with high sensitivity for different vapors [13]. The microfluidic PID consists of a serpentine channel fabricated directly on a conductive silicon wafer. A vacuum UV lamp is integrated above the microfluidic channel to ionize the analytes. The current signal generated by the PID is directly proportional to the analyte concentration. The sensor detected 5 volatile organic compounds—Benzene, Toluene, Ethylbenzene, m-Xylene, and Hexane with a LOD of 1.4 ppt, 1.2 ppt, 1.3 ppt, 1.2 ppt, and 8.8 ppt, respectively. However, a standalone PID detector is not selective since it ionizes most of the gases and produces a signal response. Hence PID sensors cannot be used as standalone detectors and must be coupled with GC systems for desired performance.

## 5. Bubble/Droplet-Based Microfluidics

In droplet/bubble-based gas sensors, the particles in the air (aerosols) are trapped within a droplet, or gases are made into bubbles. Then, the droplets/bubbles are manipulated and analyzed to detect their constituents. For example, water or oil droplets can be suspended in a continuous oil or water flow. To detect a particular gas, specific reagents are introduced into the droplets (or already present in the liquid that forms the droplets), reacting with the trapped gases or other molecules to produce a distinct color or fluorescence.

There are multiple advantages of using bubbles/droplets-based gas sensors: (1) They scale up the concentration of the analyte to be detected. Hence, even initial low concentrations of analyte can be detected. (2) Efficient use of reagents since the sample volume is small. (3) High response time since the reaction between the reagent and analyte happens rapidly inside the droplets. (4) Since the reaction occurs in small volumes, it enables the measurement of single molecules or single cells [57].

**Figure 8:**
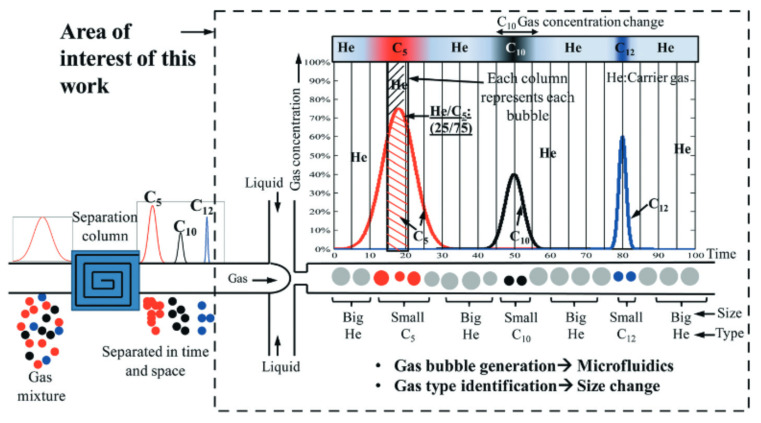
Schematic showing the working of bubble-based microfluidic gas sensor in producing gas chromatographs. Reproduced with permission from [35] Copyright 2015, The Authors, published by Royal Society of Chemistry.

Ashrafuzzaman et al. developed a bubble-based microfluidic gas sensor that analyzes the variations in bubble sizes to determine the type of gases present in the gas mixture [35]. The gas mixture is first separated in time and space into a discrete group of individual gases by a conventional chromatographic column. Then, helium (He) is used as the carrier gas to transport these separated gases into the liquid channel, where the liquid flow will cut the gas stream into a train of discrete bubbles (as shown in Figure 8). It is found that 5 types of gases (CO_2_, He, H_2_, N_2_, and CH_4_) produced unique bubble volumes of 0.44, 0.74, 1.03, 1.28, and 1.42 nL, respectively, under identical flow conditions of gas pressure and liquid flow rate. The size of bubbles is found by analyzing (using a MATLAB-based custom program) individual frames of videos shot from a video camera coupled with a microscope. By monitoring and plotting variations in bubble size, a gas chromatogram is prepared to identify the different gas molecules present in the gas mixture. This is schematically shown in Figure 8.

Triandazi et al. have developed a droplet-based microfluidic platform for detecting ammonia in a gaseous mixture [37]. The device consists of three units: Generation, collection, and harvesting. As the name suggests, the generation unit generates the liquid droplets in a continuous gaseous flow. The collection unit stores these liquid droplets in an immiscible liquid. These droplets are sent for analysis and further processing to the harvesting unit. 50% solution of Nessler’s Reagent (NR) is mixed with the water used to produce the liquid droplets. A mixture of gaseous ammonia in the air is used as the continuous phase. When the droplets were exposed to this gaseous mixture, the color of the droplets shifted due to Nessler’s reaction. By capturing the images of these droplets and converting them into 8-bit grayscale format (0–255 intensity values), every droplet is assigned an average intensity value of its constituting pixels. An appropriate intensity threshold designates the result as negative or positive.

Damit et al. have developed a droplet microfluidics-based bioaerosol detector, which could distinguish between a biological (*E. coli*) and non-biological aerosol (dried lysogeny broth particles (LB)) [36]. In this, the aerosols are aerodynamically focused into microfluidic droplets containing reagents that react with these aerosols to produce distinct fluorescence. The droplets contain propidium iodide (PI) as the reagent. As a result, the fluorescence profile produced by the droplet containing LB aerosol (non-biological) is uniform across the droplet, while *E. coli*-loaded droplets produced a profile with punctuated fluorescence distribution. This characteristic fluorescence profile can be used to distinguish between biological aerosols and non-biological aerosols.

**Figure 9:**
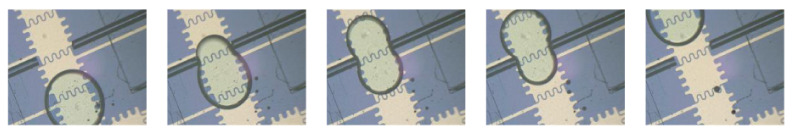
The movement of an MTB-Ba sulfate solution droplet during a measuring. Reproduced under the terms of CC BY 4.0 license from [38] Copyright 2020, The Authors, published by MDPI.

Huang et al. fabricated a digital microfluidic sensor that uses a colorimetric technique to detect various inorganic ions (sulfate, nitrate, and ammonium) in aerosols [38]. The device consists of aerosol impaction (for collection of aerosols), digital microfluidics technology that allows the manipulation of micro-droplets, and a detection part. The digital microfluidic system comprises top and bottom (with patterned electrodes) hydrophobic plates that sandwich the fluid layer. In this sensor, aerosols are collected on the surface of the digital microfluidic chip, and droplets are transported across the aerosol deposit to extract water-soluble components. The transport of the liquid droplets (shown in Figure 9) is accomplished by changing the contact angle of the liquid by changing the applied potential between two electrodes. Then, the droplets with the target are mixed with a droplet of compound-specific reagent to form a colored complex. MTB-barium complex is used as the mixing reagent to detect the sulfate concentration. As the sulfate concentration increases in mixed solution, light absorption due to the MTB-barium complex decreases, resulting in a negative relationship between sulfate concentration and absorbance. The LOD for ammonium and sulfate is 0.75 ppm and 11 ppm, respectively.

## 6. Applications

### 6.1. Air Pollutants and Particulate Matter

Nitric oxide and nitrogen dioxide are known as Nitrogen oxides (NO_x_). NOx emission into the atmosphere is caused due to the combustion of fossil fuels. NOx causes severe environmental problems such as acid rain, smog formation, and damage to the human respiratory tract. Wang et al. developed a microfluidic gas sensor with colorimetry that can measure total NOx (NO + NO2) with a detection limit of 50 ppbV [18]. Using the developed sensor, gases collected from several locations are analyzed, and it is found that the air sample of car exhaust has a very high NOx level of 1 ppmV.

Carbon monoxide is the other major pollutant that is produced due to the combustion of fossil fuels. Hossein-Babaei et al. fabricated a microfluidic filter by coating PEDOT: PSS on a microfluidic channel [11]. This allows contaminants such as hexane, benzene, and CO to pass through the channel (and get detected by the MOS sensor at the end of the channel) while methanol, ethanol, and acetone are blocked. Furthermore, the temporal response of hexane, benzene, and CO is distinct, making this gas sensor selective. Therefore, this sensor can detect low levels of CO in a highly alcohol-contaminated background atmosphere. Ammonia is an air pollutant whose emission is increasing due to various human activities. US occupational safety and Health Administration states that the recommended exposure limit to ammonia is 25 ppm, averaged over an 8 h period [58]. A microfluidic sensor for the detection of ammonia is developed by Martini et al. with a LOD of 10 ppm. It also shows a linear range between 10 to 100 ppm. The platform uses a MOS sensor with an integrated pumping system to detect ammonia.

Another important class of air pollutants is particulate matter (PM), a heterogeneous mixture of solid and liquid particles suspended in the air [59]. PM pollution is caused due to forest fires and the smoke released from factories, cars, and construction sites. PM exposure is a cause of various health problems such as irregular heartbeat, asthma, and decreased lung function [59]. Li et al. developed a virtual impactor (VI) consisting of microfluidic channels to separate fine and coarse particles [20]. An extended field effect transistor is used to detect the separated fine particles. Distinct sizes of PMs produced distinct levels of current response, which allows the sensor to give size information of the PM. Sun et al. used paper-based microfluidics and colorimetric technique to detect the presence of 6 different metals from airborne PM [19]. Cell phone photography, a self-developed iOS app, and a custom-made field reaction kit allow the on-site quantification of the 6 metal constituents found in PM.

### 6.2. Airborne Pathogens

Detecting airborne pathogens such as bacteria and viruses is essential in preventing the spread of infectious diseases. Therefore, detection techniques such as FET and fluorescence have been incorporated with microfluidic platforms to detect various bacteria and viruses.

#### 6.2.1. Bacteria

Jiang et al. developed a fluorescence-based microfluidic system to selectively detect 5 different bacteria (*S. aureus, E. coli, P. aeruginosa, C. koseri*, and *K. pneumonia*) [28]. The design of the sensor is such that it can detect the presence or absence of these bacteria in a mixture of targets. Since it is a fluorescence-based sensor, the naked eye could detect the result. The sensor showed a detection limit of 24 CFU for *S. aureus*. Such sensors have enormous potential for point-of-care applications. Choi et al. developed an opto-microfluidic platform for the continuous and real-time detection of *E. coli*, *Bacillus,* and *Staphylococcus epidermis* [25]. The Opto-microfluidic sensor achieved better detection efficiency than conventional techniques such as microscopy cell counting and colony counting.

#### 6.2.2. Viruses

Epidemics caused by transmissible respiratory viruses are a leading cause of mortality. The COVID-19 pandemic has caused more than 6.45 million deaths and affected nearly 596 million people across 200 countries as of August 2022 [60]. An efficient sensor that can detect airborne viruses in real-time is the need of the hour. Multiple microfluidic platforms have been developed for the detection of airborne viruses. Using a microfluidic paper chip, Kim et al. could detect SARS-CoV-2 from the air [26]. UV-inactivated SARS-CoV-2 is spiked into 10% v/v human saliva solution with different concentrations. 600 pg/mL is the normal concentration of SARS-CoV-2 in the human saliva of a COVID-19 patient. The LOD of the developed sensor is as low as 200 pg/mL. Similarly, Xiong et al. used LAMP primers and fluorescence in a rotating microfluidic system to detect SARS-CoV-2 with a detection limit of 10 copies/uL [27]. To detect H3N2 viruses in air Shen et al. integrated modified single silicon nanowire FET with microfluidics. The lowest concentration the authors tested is 10^4^ viruses/uL. The selectivity of the developed sensor is successfully demonstrated using the swine flu (H1N1) virus and hose allergens.

### 6.3. Gases Released from Foods

Lee et al. used a bioelectronic nose combined with a microfluidic system to detect TMA released from different food samples [8]. Olfactory receptor-derived peptides are coated on SWNT-FETs for the selective detection of TMA. Spoiled seafood generates TMA, so the amount of TMA indicates the freshness of the seafood. While analyzing gas samples from different spoiled foods, it was found that spoiled seafood had the highest response. The developed TMA sensor has a detection limit of 10 ppt, much lower than conventional TMA sensors.

Kuznetsov et al. used the fabricated microfluidic bioelectronic nose to detect vanillin in gas samples obtained from roasted coffee beans [21]. Van74 DNA aptamer, which has an affinity to vanillin, is immobilized on the ion-sensitive field-effect transistor (ISFET) for selective detection. As a result, the detection limit of the developed sensor is as low as 10 ppt.

### 6.4. Explosives

Real-time detection of explosives such as DNT is vital for homeland security and public safety applications. Coupling highly sensitive detection techniques, such as SERS, with microfluidics will help create a hand-held device that can detect trace levels of DNT. Piorek et al. developed a continuous real-time sensor by coupling free surface microfluidics with the SERS technique to create a real-time sensor that detects DNT with a LOD of 1 ppb [30]. The SERS substrate is a colloidal solution of nanoparticles that is continually refreshed using a transpirational pump [61], allowing the continuous detection of DNT.

## 7. Practical Approaches toward Using Microfluidics-Based Devices in Air

Microfluidics is an excellent candidate in scenarios where small sample volumes must be analyzed. Moreover, microfluidics helps channel the gas flow to the sensing material. It is also suitable for developing miniaturized gas sensors for POU/POC applications. Therefore, implementing microfluidics is the next logical step in developing gas sensors. We can classify these gas sensors into two broad areas (i) Single use disposable sensors and (ii) Real-time continuous sensors. Single-use sensors are suitable in applications where continuous detection is not required. The main advantage of single-use sensors is the low cost and ease of fabrication. Using paper microfluidics and colorimetric detection technique is one of the best ways to implement single-use disposable sensors. Sun et al. have used this technique to create a microfluidic sensor to detect 6 different airborne particulate matter with extremely high sensitivity [19]. Smartphone-based applications can be developed to do quantification in such colorimetric sensors. Real-time continuous detection of gases and other airborne pathogens is necessary for industrial areas and workplaces. SERS has shown enormous potential in the real-time sensing of gases and VOCs. It provides high sensitivity and selectivity with the ability to do multiplex sensing. However, to do detection in a continuous manner using the SERS technique, researchers need to regenerate the SERS substrate after each detection cycle. Laufente et al. have reported using gold-mesoporous silica nanoparticles as the SERS substrate in microfluidic gas sensors, which can be regenerated by degassing at 200 °C for 60 min [32]. However, this is not ideal since it takes a long time for complete regeneration to happen. Piorek et al. used a colloidal solution of nanoparticles as the SERS substrate in their microfluidic device, which can be continually refreshed using a transpirational pump. This ensures that the analyte always interacts with a fresh SERS substrate layer. One way to develop a continuous real-time sensor with a colorimetric technique is to couple it with bubble/droplet-based microfluidics technology. Fluorescence-based techniques have also continuously been used to detect airborne bacteria [22].

One of the components missing from closed microfluidic channel sensors is an integrated sampling system. Almost all researchers use an external pump or syringe to deliver gas samples into the microfluidic channel. To develop a fully automated miniaturized microfluidic sensor, having an integrated sampling system is particularly important. Martini et al. have developed a microfluidic gas sensor that uses the thermal creep phenomena to pull the gas sample through the microfluidic channel [4]. This is achieved by creating a temperature gradient along the microfluidic channel. Jiang et al. used a microfluidic sampling system with a staggered herringbone mixer (SHM) structure to collect airborne bacteria [28]. This is coupled with their microfluidic sensor to achieve extremely low LOD. The advantage of using open microfluidics, such as paper microfluidic sensors, is that it does not require an integrated sampling system since the microfluidic channel is already exposed to the atmosphere. Nevertheless, this will still have the limitation faced by non-microfluidic gas sensors, which is no channeled flow of gases that could bring repeatability problems.

Further developments are needed for microfluidics-based gas sensors to make it to commercial markets. New 2D materials such as MXenens [62] and organic materials such as naphthalene diimides [63] and perylene diimides [1], which are found to be promising sensing materials, need to be incorporated into microfluidics-based gas sensors to increase the sensitivity. Microfluidics-based gas sensors can be used to detect the low concentration of target analytes in low volume of gas samples which is not explored by the researchers. Due to the size of the microfluidic channels, multiple microchannels can be incorporated into a single platform (without compromising portability) to develop a sensor that can detect multiple targets with high selectivity and sensitivity.

## 8. Conclusions

This review paper has summarized the developments in microfluidic gas and airborne pathogen sensors. Integrating microfluidics with appropriate detection techniques helps develop miniaturized gas sensors with high selectivity and sensitivity. Optimizing the microfluidic channel parameters such as geometry, dimensions, and surface area is essential for developing high selectivity and sensitivity sensors. Integrating microfluidic channels (with polymer coatings) with MOS sensors instigated the ability to discriminate between different VOCs. FET-based microfluidic sensors have been developed to detect specific gases, airborne pathogens, and PM with high selectivity and sensitivity. Microfluidic sensors using the SERS technique show multi-gas detection capability with very high sensitivity. Colorimetry is an ideal technique that can be used for developing disposable microfluidic sensors. Fluorescence-based microfluidic sensors have been used for the detection of various airborne pathogens. Fluorescence and colorimetry have been coupled with bubble/droplet-based microfluidic technology to create real-time continuous sensors for detecting gases and airborne pathogens. Multiple microfluidic sensors have undergone field testing and show great potential for POU/POC applications.

## Figures and Tables

**Table 1:** Summarizes the different characteristics of microfluidic sensors developed for detecting gases or airborne pathogens.

Detection Technique	Target Analyte(s)	Transducer Material	LOD/ Lowest Concentration Tested	Selectivity	Merits	Limitations	Ref
**ELECTRONIC**							
**Metal oxide semiconductor (MOS)**	Ammonia	Tungsten trioxide film	10 ppm	Poor selectivity due to the use of MOS sensors. No techniques are employed to improve selectivity.	Integrated sampling system by thermal creep.	Poor selectivity. Need to maintain a constant working temp of 473 K	[4]
	methanol, ethanol, propanol, pentanol, hexanal, and toluene	Commercially available MOS Sensor	100 ppm	Can differentiate between 6 Volatile organic compounds (VOCs)	High surface area channel to improve selectivity. Good response and recovery time.	Fails to detect gas in gas mixture selectively.	[16]
	methanol, ethanol, 1-propanol, 2-pentanol, acetone, pentane, and hexane	Commercially available MOS Sensor (FIGARO, TGS 2602)	Not reported	Can differentiate between 7 VOCs	Channel with a coating to improve selectivity.	It needs 4 layers of coating to get good selectivity. Fails to detect gas in gas mixture selectively.	[12]
	Carbon monoxide, hexane and benzene	Commercially available MOS Sensor	Not reported	It can distinguish between carbon monoxide, hexane, and benzene. Filters out methanol, ethanol, and acetone.	Filters out ketones.	Fails to detect gas in gas mixture selectively.	[11]
**Field effect transistor (FET)**	Airborne influenza A H3N2 viruses	Silicon nanowires functionalized with influenza A H3N2 subtype antibodies	10^4^ viruses/uL	Selective against H1N1 viruses and house dust allergens.	Real-time continuous detection. Very low response time.	Absence of integrated (on-chip) sampling system.	[9]
	Trimethylamine (TMA)	Single-walled carbon nanotubes (SWNTs) functionalized with olfactory receptor-derived peptides (ORPs)	10 ppt	Response of other odorants is very low compared to TMA	Real-time sensing with high selectivity and sensitivity. Successfully used for on-site testing.	No integrated sampling system. It may not be suitable for continuous sensing.	[8]
	Silicon dioxide (SiO_2_) particles (model for PM)	Gold auxiliary electrode	Not reported	It can discriminate between 2, 5, and 10 um SiO_2_ particles.	Sensor material can differentiate between different sizes of particles.	Sensor response is highly dependent on generation velocity.	[20]
	Vanillin	Aptamer-modified ion selective FETs	2.7 ppt	The response generated by the gaseous vanillin is much stronger than that generated by the other odorant molecules.	High selectivity and sensitivity.	Very noisy response. Extensive sample preparation is needed.	[21]
**ELECTROCHEMICAL**							
**Amperometric**	Nitrogen oxide	Catalytic gold-hexacyanoferrate (Au-HCF) working electrode	~1 nM	Selective against common interfering agents (nitrite, ascorbate, ammonia, etc.)	Real-time sensing and reversible detection of NO.	Sensors are polarized for at least 4 h before use.	[22]
	Hydrogen (H_2_)	Platinum nanoparticle-modified gold microchannel electrode	3.4 vol%	No selectivity experiments were reported.	Ultra-fast response time of 2 s.	Background gases might interfere with the oxidation of H_2_	[23]
**OPTICAL**							
**Colorimetric**	Nitrogen oxide	o-Phenylenediamine (PDA)	50 ppb/min	A molybdenum oxide-based filter is highly selective against common interfering gases and ozone interference.	Prolongs the lifetime of the colorimetric sensor without sacrificing its sensitivity.	The lifetime of the sensor is still short compared to microfluidic sensors that employ other detection techniques.	[18]
	Cobalt (Co) Copper (Cu) Iron (Fe) Manganese (Mn) Chromium (Cr) Nickel (Ni)	Chrysoidine-G Dithiooxamide 1,10-phenanthroline monohydrate 4- (2-pyridylazo) resorcinol 1,5-diphenylcarbazide Dimethylglyoxime	8.16 ng 45.84 ng 1.86 × 10^2^ ng 10.08 ng 1.52 × 10^2^ ng 80.40 ng	Detection reservoirs are coated with ligands specific to the different metals	Rapid and cost-effective fabrication method—UV curing method. 48 chips made in the 30 s with a cost of $ 1.92. Use of smartphone and app for multiplex quantification.	Not a direct capture of PM particles onto the paper microfluidic chip.	[19]
	Formaldehyde	4-aminohydrazine-5-mercapto- 1,2,4-triazole (AHMT)	0.01 ppm	Unaffected by the presence of other gases such as acetaldehyde and VOCs.	Automated quantification using a smartphone-based system.	Single-use sensor.	[24]
**Fluorescence**	*Escherichia coli* *Bacillus subtilis* *Staphylococcus epidermis*	SYTO82 dye	9.9 ± 0.18/μL 7.8 ± 0.17/μL 6.5 ± 0.25/μL	Can differentiate between fluorescent bacterial cells from other residue particles.	Better detection efficiency than conventional microscopy cell counting and colony counting techniques. Gives additional information on the total particle number concentration and continuous real-time detection.	It cannot discriminate between the three bacteria.	[25]
	Severe acute respiratory syndrome coronavirus 2 (SARS-CoV-2)	Antibody-conjugated (Rabbit polyclonal antibody to SARS-CoV-2) submicron fluorescent particle (Yellow-green fluorescent carboxylated polystyrene particles).	200 pg/mL	The use of polyclonal antibodies might undermine the selectivity of the device.	Direct capture of SARS-CoV-2 virus from air.	Not a real-time detection. Needs to undergo post-sampling procedures for quantification.	[26]
	SARS-CoV-2	LAMP primer targeting the O gene and N gene	10 copies/uL	Very high selectivity. 16 other viruses showed negative results.	Clinically verified.	Not a real-time detection. Needs to undergo post-sampling procedures for quantification.	[27]
	*S. aureus, E. coli, P. aeruginosa, C. koseri, and K. pneumonia*	Separate LAMP primers targeting each bacterium	24 colony-forming units (CFU) for *S. aureus*	High selectivity because of the use of the LAMP technique.	Capable of detecting 5 different bacterial species. The results generated are visible to the naked eye.	We need to incubate the chip at 63 °C for 50 min. Not a real-time detection. Only qualitative analysis.	[28]
**Surface-enhanced Raman spectroscopy**	4-aminobenzenethiol(4-ABT)	The colloidal suspension of silver nanoparticles	300 uM	Highly selective	Fast response time (1–3 s).	Sensors can only detect the water-soluble analyte.	[29]
	2,4-dinitrotoluene (DNT)	Colloidal solution of silver nanoparticles	1 ppb	Highly selective	Continuous detection of the analyte. Quick response time (t ≈ 2 min)	Experiments performed under fixed relative humidity (40% RH)	[30]
	4-methylbenzenethiol (4-BMT)	Glass + Au microstructures	0.5 M 4-MBT	Highly selective	Direct metal writing using two-photon lithography fabricates microfluidic channels with densely packed AuNP. Very low response time (2 s).	Use of expensive instruments for fabrication of sensors.	[31]
	Dimethyl methylphosphonate (DMMP)	Gold-mesoporous silica nanoparticles	2.5 ppmV	Highly selective	Regeneration of SERS substrate is possible by desorption at 200 °C.	Complete regeneration of substrate takes 60 min.	[32]
	Benzaldehyde 3-ethylbenzaldehyde glutaraldehyde	Zeolitic Imidazolate framework-8 (ZIF-8) and cysteamine (CA)-coated silver nanotubes (AgNCs)	1 ppb 1 ppb Not reported	High selectivity for aldehydes. Acetone, ethanol, and toluene showed very low SERS intensity.	Very high sensitivity and selectivity.	No reusability test is reported.	[15]
	DNT Odoriferous benzaldehyde Indole	Polystyrene microspheres with nano pits containing bimetallic nanocubes (gold core of 30 nm, silver nanocube of 54 nm, and a 2 nm gold shell)	10 ppb 10 ppb 50 ppb	Highly selective	Excellent multiplexing ability with high sensitivity.	Elaborate sample preparation is required.	[33]
**Absorbance spectrum**	Ammonia	Ninhydrin—PDMS composite	2 ppm	Highly selective	Excellent response time (5–10 s)	We may need to replace the sensing material after every run. Not suitable for continuous sensing.	[34]
**Photoionization**	Benzene Toluene Ethyl benzene m-Xylene Hexane	Two electrodes	1.4 ppt 1.2 ppt 1.3 ppt 1.2 ppt 8.8 ppt	No selectivity	Excellent sensitivity. The ideal candidate is the detector in gas chromatography systems.	It cannot be used as a standalone gas sensor.	[13]

**Table 2:** Summarizes the different characteristics of bubble/droplet-based microfluidics for detecting gases or airborne pathogens.

Bubble/Droplet-Based Techniques							
Detection Technique	Target Analyte	Transducer Material	LOD/Lowest Concentration REPORTED	Selectivity	Merits	Limitations	Ref
**Bubble-based**	Carbon dioxide (CO_2_) Helium (He) Hydrogen gas (H_2_) Nitrogen gas (N_2_) Methane (CH_4_)	No transducer material. Detection based on the size of individual bubbles	Not reported	Can selectively detect 5 different gases (CO_2_, He, H_2_, N_2,_ and CH_4_)	It can be used for continuous real-time detection.	Uses a conventional chromatographic column.	[35]
**Fluorescence**	*Escherichia coli* (*E. coli*)	Droplets containing propidium iodide (PI.)	Not reported	Can differentiate between non-bioaerosol and bioaerosol	Detection happens within 20 s.	Not portable due to the use of a conventional fluorescence microscope	[36]
**Colorimetric**	Ammonia	Nessler’s reagent	500 ppm	Selectively detects ammonia	Suitable for continuous real-time detection	Poor sensitivity.	[37]
	Sulfate Ammonium	Methylthymol blue (MTB)	11 ppm 0.256 ppm	The reaction is selective.	Sampling, manipulation, and detection are integrated into a single chip.	Complex fabrication procedure.	[38]

## Data Availability

Not applicable.
